# Study on the Effects of Dandelion Ultrafine Powder on the Production Performance and Intestinal Microbiota of Broiler Chickens

**DOI:** 10.3390/ani16203264

**Published:** 2026-10-16

**Authors:** Qingshu Tao, Ziqi Chen, Haifang Liu, Shuqing Li, Jian Ma, Jiaping Zhou, Yun Yan, Yingqi Liu, Yong Wang

**Affiliations:** 1 College of Agricultural Science and Technology, Shandong Agriculture and Engineering University, Jinan 250100, China; tqs@sdaeu.edu.cn (Q.T.); ngylishuqing@163.com (S.L.); z2013490@sdaeu.edu.cn (J.M.); jiaping1022@163.com (J.Z.); yanyun_yyyy@163.com (Y.Y.); liuyingqi428@163.com (Y.L.); 2 Tianjin Key Laboratory of Agricultural Animal Breeding and Healthy Husbandry, College of Animal Science and Veterinary Medicine, Tianjin Agricultural University, Tianjin 300392, China; chenzq0601@163.com; 3 Wenshang Animal Husbandry and Veterinary Development Center, Wenshang 272500, China; liuhaifang2005@126.com

**Keywords:** dandelion ultrafine powder, broilers, production performance, intestinal microbiota

## Abstract

This study aimed to investigate the effects of dietary supplementation with dandelion superfine powder (DSFP) on the growth performance, organ indices, intestinal length, antioxidant parameters, cecal microbiota and economic benefits of broilers, so as to determine its optimal dosage. A total of 240 one-day-old ROSS broilers were randomly allocated into four groups fed basal diets supplemented with 0%, 0.5%, 1% and 3% DSFP, respectively. The results showed that medium-dose (1%) DSFP significantly increased the overall ADG and average daily feed intake, elevated duodenal length, and improved serum GSH-Px activity of broilers. Microbiological analysis revealed that 1% DSFP was associated with changes in the cecal microbiota structure and tended to increase the relative abundance of Firmicutes and potential short-chain fatty acid-producing bacteria. Economically, the medium-dose group achieved the highest gross profit per broiler. In conclusion, dietary supplementation with 1% DSFP can effectively improve growth performance and was associated with favorable changes in certain intestinal microbial indicators, leading to superior economic benefits, and it represents the appropriate dosage for broiler production.

## Simple Summary

The overuse of antibiotics in poultry production has led to bacterial resistance and drug residues, threatening food safety and public health. As a result, safe and effective antibiotic alternatives are urgently needed. Dandelion (*Taraxacum mongolicum* Hand.-Mazz.) is an edible and medicinal plant rich in bioactive compounds, but its high crude fiber content limits its utilization. In this study, dandelion was processed into dandelion ultrafine powder (DSFP) and added to broiler diets at 0%, 0.5%, 1%, and 3% to evaluate its effects on growth performance, organ development, intestinal length, antioxidant capacity, cecal microbiota, and economic benefits. The results showed that 1% DSFP supplementation produced the best overall effects: it significantly increased ADG and average daily feed intake, promoted duodenal development, enhanced serum glutathione peroxidase (GSH-Px) activity, and was associated with changes in the cecal microbiota, including a tendency toward a higher relative abundance of Firmicutes and potential short-chain fatty acid-producing bacteria, and achieved the highest gross profit per bird. These findings indicate that 1% DSFP is an appropriate dosage for broiler production and may serve as a promising natural antibiotic alternative.

## 1. Introduction

For decades, antibiotics have been widely applied as feed additives in livestock and poultry production to prevent disease, improve growth performance, and reduce feed costs [1,2]. However, the widespread and extensive overuse of antibiotics not only induces bacterial resistance but also generates antibiotic residues [3,4]. Ultimately, these hazardous residues transmit through the food chain to humans and pose severe threats to public health [3]. Globally, an increasing number of countries have banned the addition of antibiotic additives in livestock and poultry feeds [5].

Commercial broilers grow rapidly, yet their visceral organs—especially the intestinal tract—fail to develop synchronously with their fast body growth. This developmental imbalance readily induces oxidative stress and intestinal microbiota dysbiosis in broilers [6]. Accordingly, exploring jointly confirmed low-cost, efficient antibiotic alternatives is critically urgent for the sustainable development of livestock and poultry breeding [1].

Following China’s official ban on antibiotic feed additives, the search for antibiotic substitutes has become extremely urgent. Traditional Chinese herbal medicines possess inherent advantages as antibiotic alternatives and have been extensively applied in livestock and poultry production [7]. Dandelion, commonly known as dandelion or yellow-flowered dandelion, is a perennial herb of the genus Taraxacum within the Asteraceae family [8]. Dandelion is an edible medicinal plant abundant in dietary fiber, minerals, amino acids, vitamins and trace elements. It also contains multiple bioactive compounds including terpenoids, polysaccharides, phytosterols and polyphenols. In China, wild dandelions are widely distributed across Northeast, North and Northwest China, and can be utilized as both edible wild vegetables and traditional Chinese medicinal materials [9]. In 2012, the Ministry of Health of the People’s Republic of China listed dandelion in the Catalogue of Items Both Edible and Medicinal, making it a vital homologous plant in China. From the perspective of traditional Chinese medicine (TCM), dandelion exerts diuretic, heat-clearing, detoxifying, detumescent, mass-dissipating, hepatoprotective, cholagogic, stomachic and digestive effects [10]. Modern pharmacological studies further confirm that dandelion possesses antioxidant, antiviral, anti-tumor, lipid-lowering, hypoglycemic, antibacterial and anti-inflammatory capacities, alongside inhibitory activity against melanin synthesis [11]. Accordingly, the application of dandelion as a feed additive has attracted growing research attention [12].

Nevertheless, raw dandelion contains high levels of crude fiber and exhibits rigid mechanical texture. Conventional crushing techniques fail to fully refine its crude fiber to achieve fine, palatable powder [12]. Ultrafine grinding technology is an emerging material processing approach that breaks the internal bonding force of raw materials via mechanical or fluid dynamic impact, grinding and shearing to prepare micron-sized particles. Ultrafine powder features tiny particle size, high dispersibility, large specific surface area, strong adsorption capacity, high dissolution rate of active substances, elevated digestibility and absorption rate, as well as superior bioavailability. At present, this technology has been widely adopted in food, pharmaceutical and chemical industries. Processing dandelion into ultrafine powder by ultrafine grinding technology facilitates the release of its bioactive components. In the present trial, dandelion was processed into ultrafine powder and supplemented into broiler diets. This study determined the growth performance, organ indices, intestinal length, antioxidant parameters, intestinal microbiota and production economic benefits of broilers, aiming to provide theoretical support for the application of dandelion superfine powder (DSFP) in broiler breeding.

## 2. Materials and Methods

### 2.1. Chickens

The protocol for the chickens in this study was approved by the Shandong Agriculture and Engineering University Institutional Animal Care and Use Committee (SDAEULLSC202501). A total of 240 one-day-old healthy ROSS 308 male broilers with no clinical symptoms or physical deformities were selected for this experiment. The average initial body weight of the chicks was 42.36 ± 0.52 g and was consistent among all individuals before grouping. All broilers were individually weighed and sorted by body weight prior to grouping. Subsequently, the chickens were evenly and randomly assigned to four dietary treatment groups using a completely randomized design to ensure no significant differences in initial body weight across groups. Each treatment group consisted of 6 biological replicates, with 10 broilers per replicate. The control group (CON) was fed with basic diet, while the low-dose group (L), medium-dose group (M) and high-dose group (H) were fed with basic diets supplemented with DSFP at 0.5%, 1.0%, and 3.0% (*w*/*w*), respectively. DSFP was prepared exclusively from the aerial parts of dandelion, namely the stems, leaves, and flowers, and the roots were not used. DSFP was added on top of the basal diet without replacing any feed ingredient. The composition of isoenergetic and isoprotein basal starter and grower diets given to the experimental chickens is shown in Table 1.

All experimental chickens had free access to feed and water throughout the trial. The experiment was carried out in a ventilated indoor poultry house with a raised wire-mesh floor. Each pen covered an effective area of 2.5 m^2^ (2.0 m × 1.25 m), with a standardized stocking density of 4 birds/m^2^ in accordance with ROSS 308 rearing guidelines. The room temperature was maintained at 33–34 °C during the initial 3 days and gradually reduced to 22–24 °C at 21 days of age. The lighting program was set as 23L:1D (20 lx) for the first week and adjusted to 16L:8D (20 lx) for the remaining experimental period. Manure beneath the wire mesh was cleaned twice weekly to ensure a dry rearing environment. All broilers were vaccinated following standard commercial procedures: ND (Dahuanong Animal Health, Zhaoqing, China) vaccine via nasal and eye-drop administration at d 7, IBD vaccine via drinking water at d 14, ND booster immunization at d 21, and IBD (Dahuanong Animal Health, Zhaoqing, China) booster immunization at d 28. No antibiotics or additional vaccines were applied during the experiment.

### 2.2. Sample Harvest

The chickens were weighed on an empty stomach every Monday morning at 8:00, starting from the beginning of the experiment, and the animal feed intake was recorded.

At 42 days of age, the chickens were fasted for 12 h. One chicken was randomly selected from each group, blood was collected from the wing vein and serum was separated and stored in a refrigerator at −20 °C for future use. Then, the chickens were anesthetized with sodium pentobarbital (Pentobarbital sodium Cat. No. P3761, Sigma-Aldrich, St. Louis, MO, USA) (30 mg/kg BW) via wing vein injection. The depth of anesthesia was confirmed by loss of corneal reflex, loss of pedal reflex, and no response to painful stimuli. After confirming adequate anesthesia, euthanasia was performed by exsanguination via the jugular vein. Death was confirmed by cessation of heartbeat, cessation of respiration, dilated pupils, and loss of corneal reflex. Bilateral pectoral and leg muscles, as well as abdominal fat, were collected and weighed. The peeled pectoral and leg muscles were packaged in resealable bags. A portion of the meat samples was stored at 4 °C, while the remaining samples were stored at −80 °C for future use.

### 2.3. Measurement of Growth Performance

The determination of relevant production performance was carried out in accordance with poultry production performance terminology and measurement statistical methods [13] (NY/T 8232020). Broilers were weighed on an empty stomach at 08:00 each morning every week during the experiment, and feed intake was recorded. Average daily gain (ADG), average daily feed intake (ADFI) and feed conversion ratio (FCR) were calculated.

The calculation formula is as follows:

ADG (g/d) = (Final body weight − Initial body weight)/Number of feeding days

ADFI (g/d) = Total feed intake during the experimental period/Number of feeding days

FCR = Total feed intake during the experimental period/Total body weight gain during the experimental period

### 2.4. Intestinal-Related Indicators

Total microbial DNA was extracted from fresh cecal contents homogenized in sterile PBS using the E.Z.N.A.^®^ Soil DNA Kit (Omega Bio-tek, Norcross, GA, USA). DNA quality was verified by 1% agarose gel electrophoresis, while DNA concentration and purity were determined with a NanoDrop 2000 spectrophotometer (Thermo Scientific, Waltham, MA, USA). The V4 hypervariable region of the 16S rRNA gene was amplified with universal primers specifically designed for gut microbial community analysis. PCR amplicons were purified using the Omega DNA Purification Kit (Omega Bio-tek, Norcross, GA, USA), and sequenced on the Illumina MiSeq platform (Illumina Inc., San Diego, CA, USA).

For bioinformatic processing, raw sequencing reads were quality-filtered to remove low-quality sequences and paired-end reads were merged. High-quality sequences were clustered into operational taxonomic units (OTUs) at a similarity threshold of >97% using USEARCH (version 10.0), and representative sequences for each OTU were selected under default parameters. Taxonomic assignment was performed in QIIME2 (version 2023.9) using a naive Bayes classifier against the SILVA 138.1 database with a confidence threshold of 70%. Microbial community composition was further analyzed on the QIIME2 platform (https://qiime2.org (accessed on 28 May 2026)) combined with Perl scripts. Alpha-diversity indices were calculated to assess within-sample species complexity. Principal coordinate analysis (PCoA) was conducted for beta diversity to explore inter-sample community dissimilarities. One-way ANOVA was applied for statistical comparisons of bacterial relative abundance and diversity indices among experimental groups. The linear discriminant analysis effect size (LEfSe) algorithm was used to identify taxa with differential abundance across groups [14].

### 2.5. Measurement of Slaughter Performance and Organ Index

Both pectoral and leg muscles, as well as abdominal fat, were removed and weighed to calculate the dressing percentage, eviscerated carcass weight percentage, pectoral muscle percentage, leg muscle percentage, and abdominal fat percentage. Simultaneously, the hearts, livers, spleens, lungs, kidneys, duodenum, jejunum, and ileum of the broilers were removed, weighed, and straightened. The lengths of each intestinal segment were measured using a tape measure to calculate the organ index and relative intestinal length.

The calculation formula is as follows:

Organ index = Fresh weight of organ/Live body weight; Relative length of intestine = The length of each intestine/Live body weight

### 2.6. Test Kit

The reagent kits were all purchased from Nanjing Jiancheng Biological Engineering Research Institute Co., Ltd. (Nanjing, China). The test kit mainly included antioxidant indicators (malondialdehyde (MDA), total antioxidant capacity (T-AOC), catalase (CAT), antioxidant enzymes such as superoxide dismutase (SOD), glutathione (GSH) and glutathione peroxidase (GSH-Px)). All tests were conducted according to the instructions of the reagent kit.

### 2.7. Economic Analysis

Feed price was based on the actual market prices of the basal diet (CNY/kg) and the DSFP additive (CNY/kg). Total feed cost per bird was calculated as total feed intake multiplied by feed price. Sales revenue per bird was calculated as final body weight multiplied by the market price of live birds (CNY/kg). Gross profit per bird was calculated as sales revenue minus total feed cost. The equations are as follows:

Total feed cost = Total feed intake × Feed price

Sales revenue = Final body weight × Market price of live birds

Margin over feed cost = Sales revenue − Total feed cost

### 2.8. Statistics and Analysis

All experimental data were analyzed using SPSS 20.0 (IBM Corporation, Armonk, NY, USA). Before one-way ANOVA, the Shapiro–Wilk test was used to check residual normality, and Levene’s test was applied to evaluate homogeneity of variance; data violating these assumptions were log-transformed prior to analysis. Data were first tested for normality and homogeneity of variance. Differences between groups were tested using one-way ANOVA. Growth performance indices (ADG, ADFI and FCR) were analyzed separately for 1–21 d, 22–42 d and the whole experimental period (1–42 d). The experimental unit for growth performance, organ indices, intestinal length, serum antioxidant indicators and economic parameters was each replicate pen, while one randomly selected broiler per pen was used as the sampling unit for slaughter performance, serum biochemistry and cecal microbiota measurements. When significant differences were found, Duncan’s multiple comparison method was further used for analysis. For microbiome data, alpha diversity indices and taxonomic relative abundance were compared using one-way ANOVA with Benjamini–Hochberg FDR correction for multiple comparisons. Beta diversity was assessed via PCoA based on Bray–Curtis distance, and ANOSIM was performed to evaluate overall community dissimilarity. LEfSe analysis was used to identify differential microbial biomarkers with an LDA threshold of 2, and LEfSe built-in multiple testing correction was adopted. The results were expressed as “mean ± standard deviation”, with *p* < 0.05 indicating significant differences and *p* < 0.01 indicating highly statistically significant differences.

## 3. Results

### 3.1. The Effects of DSFP on ADG, ADFI and FCR of Broilers

The ADG, ADFI and FCR of broilers in each treatment group at different growth stages are presented in Table 2. During 1–21 d, broilers fed medium-dose DSFP diet (DSFP-M) exhibited significantly higher ADG than birds in the CON (*p* < 0.001), DSFP-L (*p* = 0.045) and DSFP-H (*p* < 0.001) groups. The ADG of DSFP-L group was significantly greater than that of the CON (*p* = 0.014) and DSFP-H (*p* = 0.006) groups. In the grower phase (22–42 d), the DSFP-M group obtained remarkably higher ADG compared with the CON (*p* < 0.001) and DSFP-L (*p* = 0.034) groups, and the DSFP-H group also obtained significantly higher ADG compared with the CON (*p* = 0.002) and DSFP-L (*p* = 0.050) groups. For the whole experimental period (1–42 d), the ADG of broilers increased first and then slightly decreased with the rising supplemental level of DSFP. The DSFP-M group had the highest overall ADG, which was significantly higher than the CON (*p* < 0.001) and DSFP-L (*p* = 0.030) groups. The DSFP-H group had an ADG similar to that of the DSFP-M group and a significantly superior growth performance when compared with the control group (*p* = 0.006). The DSFP-L group showed significantly higher overall ADG than the CON group (*p* = 0.036), and the control group had the lowest ADG across all treatments.

For the whole 1–42 d period, the DSFP-M group had significantly higher ADFI than the CON (*p* = 0.029), DSFP-L (*p* = 0.022) and DSFP-H (*p* = 0.029) groups, while no difference was found between low/high-dose groups and control.

Statistical analysis indicated that dietary DSFP supplementation failed to significantly improve the feed conversion efficiency of broilers at any growth stage (*p* > 0.05).

### 3.2. The Effect of Dandelion Ultrafine Powder on Organ Indexes

As shown in Table 3, the heart index of the DSFP-L group was significantly higher than that of the DSFP-H group (*p* = 0.044), whereas the kidney index of the CON group was also significantly higher than that of the DSFP-H group (*p* = 0.042).

### 3.3. The Effect of Dandelion Ultrafine Powder on the Length of the Small Intestine of Broiler Chickens

As shown in Table 4, duodenum length of the three DSFP groups was significantly higher than the CON group (*p* < 0.001). For jejunum length, the CON group showed the maximum value, which was significantly higher than the DSFP-H group (*p* = 0.026). In addition, the CON group had a significantly longer ileum length compared with the DSFP-L (*p* = 0.010) and DSFP-H (*p* < 0.001) groups, and the ileum lengths of the DSFP-L (*p* = 0.002) and DSFP-M (*p* < 0.001) groups were both longer than that of the DSFP-H group.

### 3.4. The Effect of Dandelion Ultrafine Powder on Serum Antioxidant Activity in Broiler Chickens

As shown in Table 5, No significant differences were observed among the groups regarding CAT, T-AOC, SOD, and MDA levels (*p* > 0.05). However, GSH-Px activity in the DSFP-L (*p* = 0.028) and DSFP-M (*p* = 0.026) groups was significantly higher than that in the DSFP-H group. As for GSH content, the DSFP-M group exhibited significantly higher levels compared with the CON (*p* = 0.020) and DSFP-H (*p* = 0.008) groups, and the DSFP-L group also showed significantly higher GSH levels than the DSFP-H group (*p* = 0.047).

### 3.5. The Effect of Dandelion Ultrafine Powder on Cecum Microorganism

**Figure 1:**
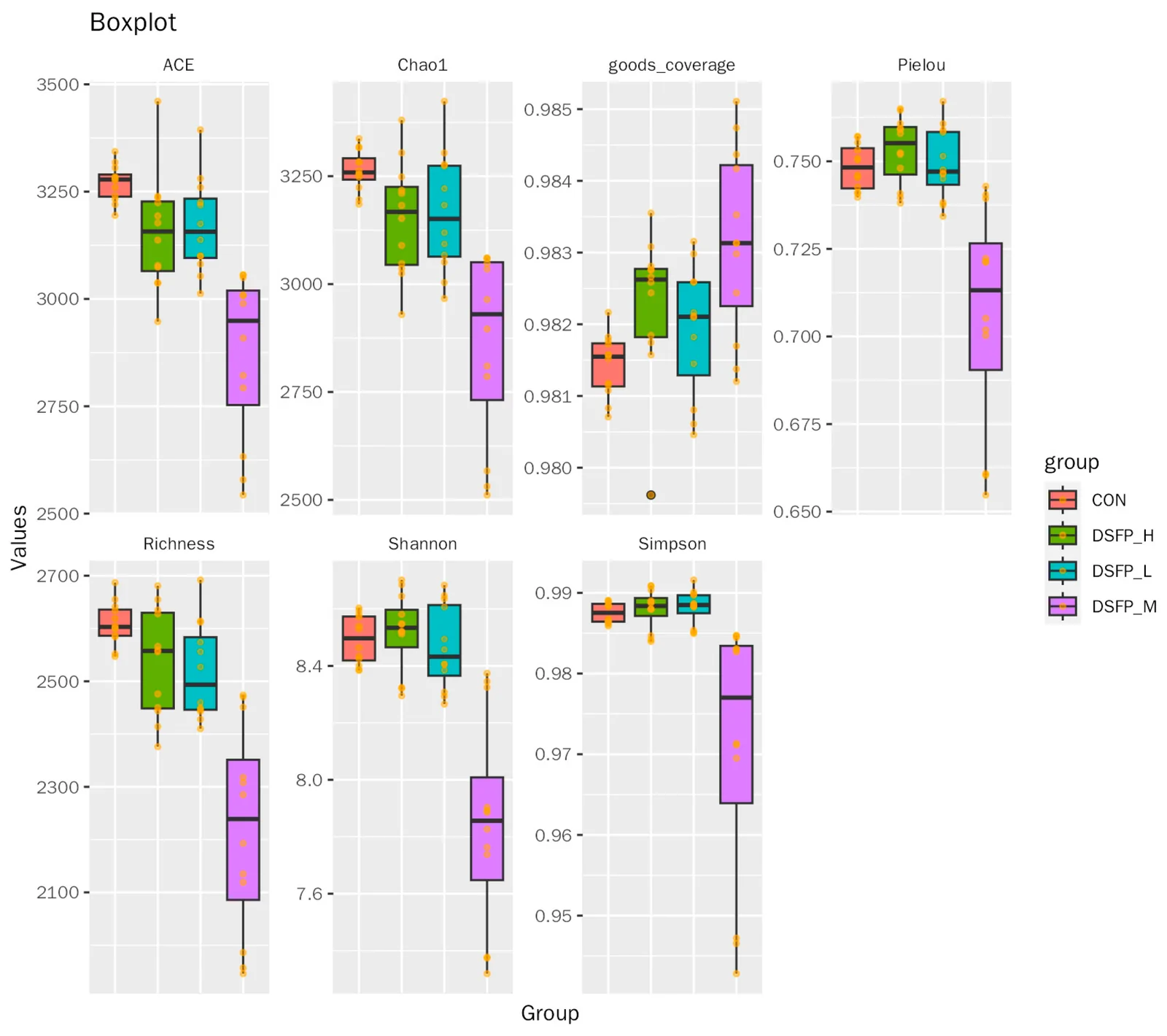
Alpha diversity indices of cecal microbiota in broilers fed diets supplemented with DSFP. The seven subgraphs represent ACE, Chao1, Goods_coverage, Pielou, Richness, Shannon and Simpson indices, respectively.

**Figure 2:**
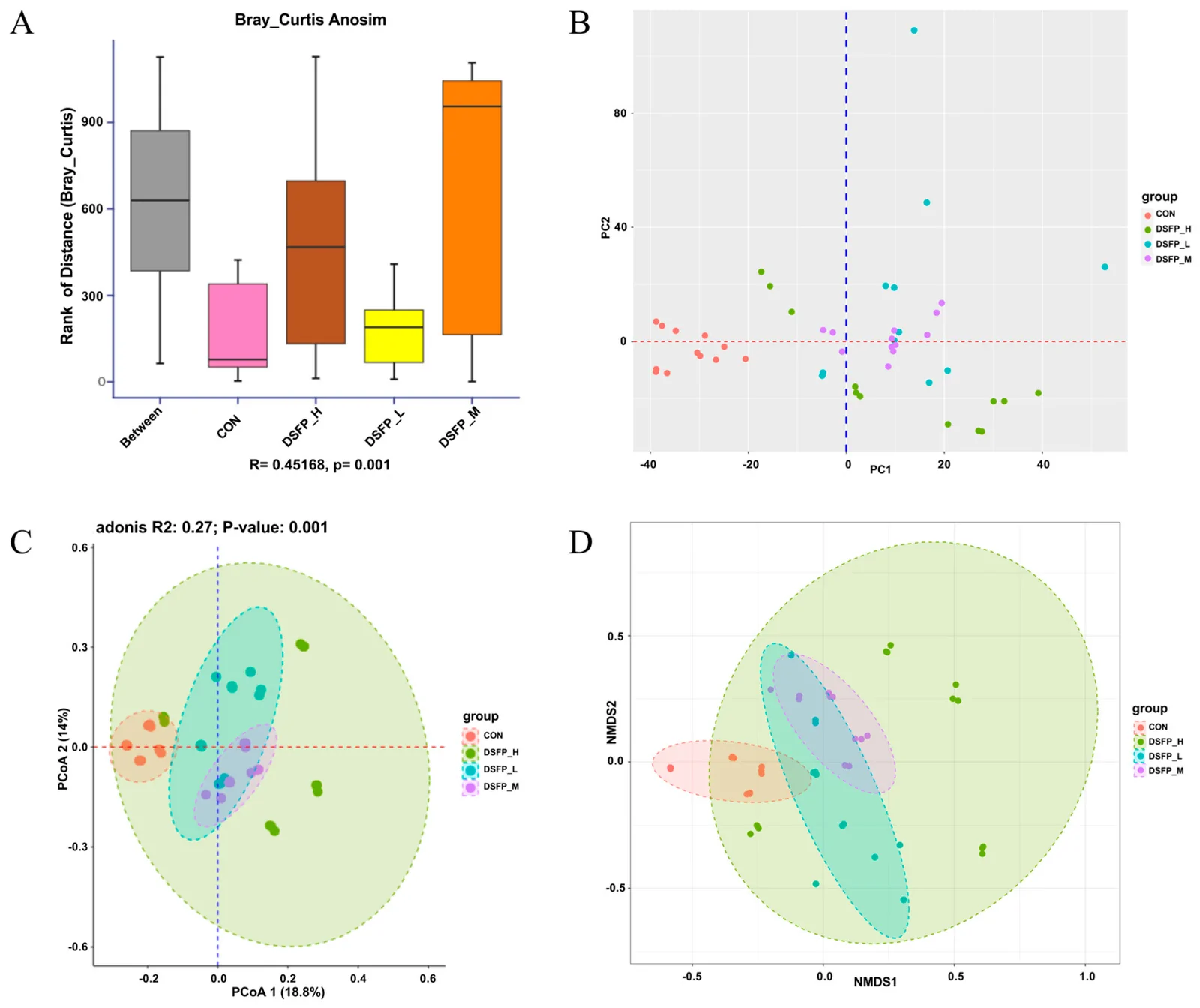
Beta diversity analysis of broiler cecal microbiota based on Bray–Curtis distance. (**A**) ANOSIM test of inter-group and intra-group community dissimilarity; (**B**–**D**) PCoA ordination plots illustrating microbial community separation among four groups.

**Figure 3:**
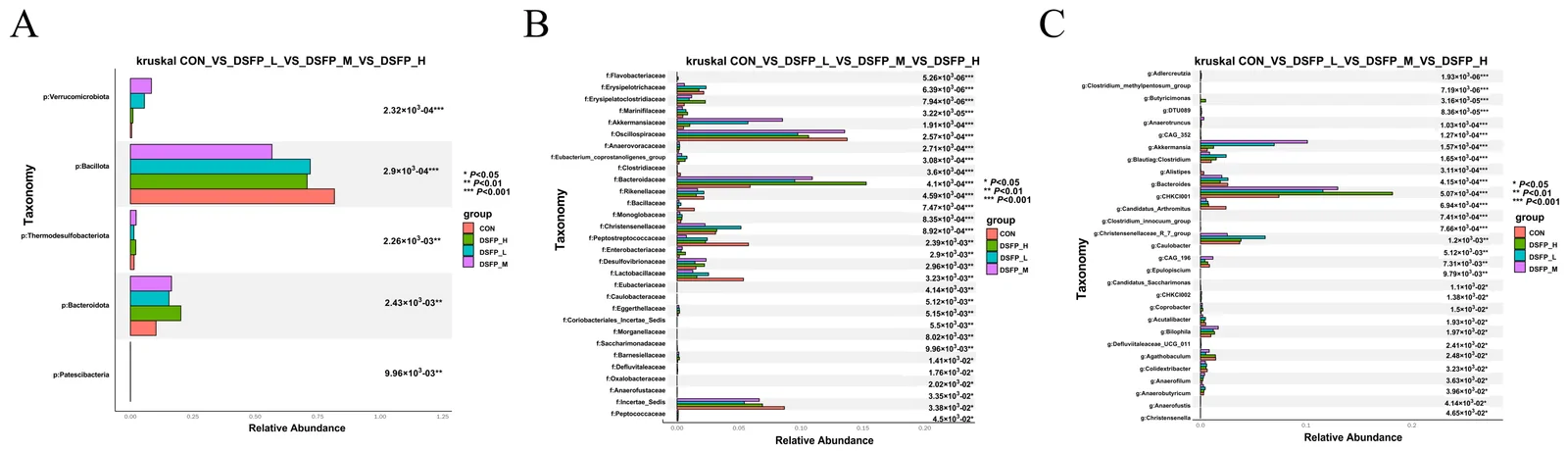
Relative abundance of dominant cecal bacterial taxa at different taxonomic levels: (**A**) phylum level, (**B**) class level, and (**C**) genus level. The horizontal axis represents relative abundance, and the vertical axis lists classified bacterial taxa. * *p* < 0.05, ** *p* < 0.01, *** *p* < 0.001. Values with different asterisks indicate significant differences between groups.

**Figure 4:**
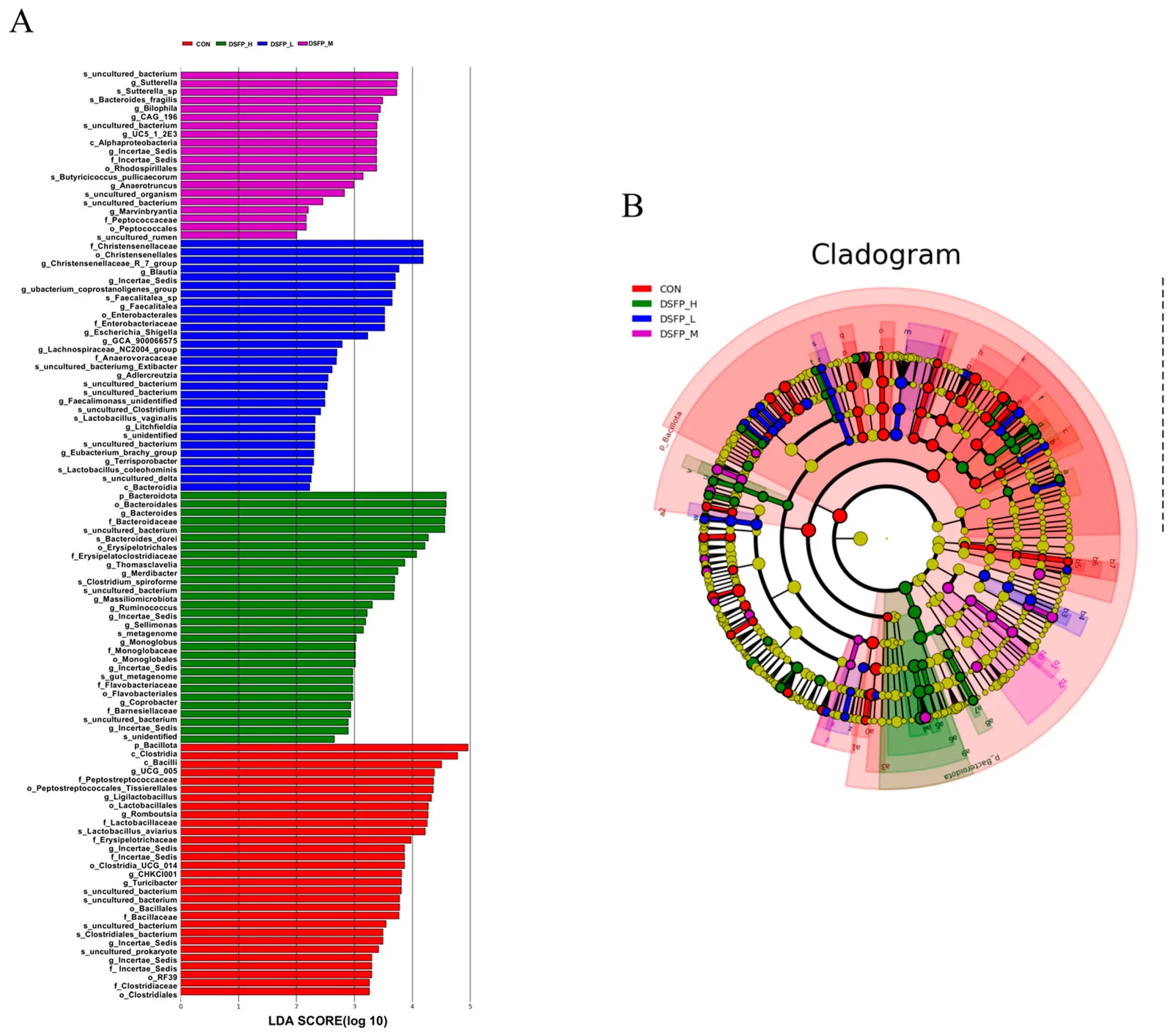
LEfSe linear discriminant analysis (LDA) of differential biomarker bacteria in each group. (**A**) LDA score histogram showing taxa with LDA > 2 and (**B**) evolutionary cladogram of differential microbial biomarkers.

The effects of DSFP on cecal microbial diversity of broiler chickens are displayed in Figure 1, Figure 2, Figure 3 and Figure 4.

Goods_coverage of all groups exceeded 0.98, indicating sufficient sequencing depth to capture most microbial species. CON, DSFP-L and DSFP-H exhibited comparable ACE, Chao1, Richness, Shannon and Simpson indices, while DSFP-M group showed markedly reduced richness and diversity values. The Pielou index was significantly decreased in the DSFP-M group relative to other treatments, suggesting that the medium-dose DSFP reduced the evenness of cecal bacterial community.

The ANOSIM test showed R = 0.4298, *p* = 0.3881, indicating no statistically significant overall difference in microbial community structure among the four treatments. The PCoA plot showed partial overlap of confidence ellipses for the CON, DSFP-L and DSFP-H groups. DSFP-M samples displayed visual separation from the other three groups, suggesting a trend of shifts in cecal microbiota composition under medium-dose DSFP treatment, although this separation was not supported by significant overall community-level differences.

Firmicutes and Bacteroidota dominated the cecal microbiota at phylum level across all groups. Compared with CON, DSFP-M significantly elevated the relative abundance of Firmicutes while reducing Bacteroidota proportion. At class and genus levels, DSFP supplementation remodeled the abundance of core bacterial genera, among which medium-dose DSFP exerted the most pronounced regulatory effect on dominant bacteria. Multiple differential genera exhibited highly significant intergroup variation (*p* < 0.001).

Each treatment group possessed unique microbial biomarkers. The CON group was enriched with multiple Bacteroidota-related taxa; DSFP-L and DSFP-H shared partial differential Firmicutes species; the DSFP-M group owned the maximum number of specific enriched biomarkers, mainly taxa previously reported as potential short-chain fatty acid-producing Firmicutes, which indicated that medium-dose DSFP selectively enriched these characteristic intestinal bacteria.

### 3.6. Effects of DSFP on the Production Economic Performance of Broilers

As shown in Table 6, broilers in the DSFP-M group exhibited greater gross profit compared with the CON group. Total feed cost per bird was significantly higher in DSFP-M compared with CON (*p* = 0.002) and DSFP-L (*p* = 0.006), and in DSFP-H compared with CON (*p* < 0.001) and DSFP-L (*p* = 0.002). Sales revenue of DSFP-M (*p* = 0.017) and DSFP-H (*p* = 0.034) was significantly greater than CON. The DSFP-M group obtained the highest gross profit per broiler, which was markedly higher than the control (*p* = 0.034).

## 4. Discussion

Although antibiotics are beneficial for the production performance and disease prevention of livestock and poultry, their contribution to antimicrobial resistance and their threat to public health are increasingly recognized, and an increasing number of countries are banning the use of antibiotics as feed additives [1]. Dandelion possesses biological activities including antioxidant, lipid-lowering, anti-inflammatory effects and intestinal microbiota regulation, all of which can improve the production performance of broilers [12]. Chinese medicine and its extracts have been shown to improve the health status and production performance of poultry [15]. A previous study showed that adding 1.0% dandelion to the diet had no effect on the growth performance of broiler chickens [16]. The most likely reason for this is that most active substances in dandelion are trapped within the cell wall, and only a small fraction of these compounds can be absorbed and utilized by broiler chickens [17]. Dietary DSFP supplementation significantly increased the ADG of broilers in a dose- and stage-dependent manner, with medium-dose DSFP showing optimal growth-promoting effects throughout the whole feeding cycle. However, it should be noted that the 1.0% DSFP treatment increased ADG while also increasing ADFI, whereas FCR did not differ significantly among treatments. Therefore, the improvement in body weight gain should be interpreted primarily as feed intake-driven growth rather than as an improvement in feed efficiency. A previous study showed that supplementing the diet with fermented dandelion at a dose of 1% could increase ADG [17], which is consistent with the present results. This may be attributed to the fact that fermentation and superfine grinding may facilitate the release of the active components of dandelion, as reported in previous literature. However, as no conventional dandelion powder control group was set in the present trial, we cannot directly confirm that ultrafine grinding improves the bioavailability of dandelion bioactive compounds. Dandelion contains abundant flavonoids, chlorogenic acid and polysaccharides, which enhance nutrient digestibility and body weight gain by alleviating intestinal oxidative stress and mucosal inflammation as well as enriching beneficial cecal microbiota. DSFP only improved ADG during the 1–21 d starter phase, because positive effects were observed on starter broilers and the immature intestines of young chicks respond to trace bioactive compounds to relieve post-hatch stress, while the mature intestines of broilers over 22 d with stronger inherent antioxidant and immune capacities cannot gain persistent growth benefits from insufficient low-dose active ingredients. The high-dose DSFP merely elevated ADG during the 22–42 d grower period without positive effects on starter broilers, which is hypothesized to occur because excessive bioactive components slightly suppress epithelial proliferation in underdeveloped chick intestines and counteract the promotion of nutrient absorption; mature intestinal tracts tolerate high DSFP levels to exert antioxidant and microbiota-modulating functions, yet the overall growth benefit of the high dose was inferior to that of medium supplementation, indicating an effective dosage threshold for DSFP. The medium dose of DSFP achieves balanced bioactivity without insufficient efficacy at low doses or intestinal irritation induced by excessive addition, matching the intestinal physiological characteristics of broilers across all growth stages, which may serve as a promising natural alternative to antibiotics in broiler production.

Early-stage phased intervention did not alter the feed intake of broilers, which may be attributed to the immature gastrointestinal development of newly hatched chicks. We hypothesize that the medium dose of DSFP exerted a feed-promoting effect and continuously enhanced the feeding motivation of broilers, possibly because the low dose of DSFP contained insufficient active components to effectively activate the central and peripheral appetite-regulating pathways. In contrast, it is speculated that the high dose of DSFP may have activated satiety-related negative feedback, thereby counteracting its feed-promoting activity.

The redox state of the body is an important indicator of its health status, and livestock and poultry maintain redox homeostasis through the endogenous antioxidant enzyme system. Oxidative stress can cause structural damage to cells, disrupt cell membranes and even organelles, affect the oxidative modification of biomolecules such as proteins, lipids, and DNA, interfere with signaling pathways, lead to metabolic disorders, reduce immunity, and trigger aging, inflammation, and chronic diseases [18]. The growth process of chickens may be influenced by oxidative stress triggered by environmental factors, disease factors, and even nutritional factors [19]. The antioxidant function of dandelion has been confirmed in laying hens [12] and Luhua chickens [20]. SOD and GSH are key antioxidant enzymes that protect cells from oxidative damage [12]. SOD has a certain alleviating effect on inflammation by inhibiting the excessive accumulation of free radicals, reducing the oxidative damage of free radicals to DNA and proteins, and reducing oxidative stress to support immune cell activity [21]. GSH-Px specifically catalyzes the reduction reaction of GSH to hydrogen peroxide and other lipid peroxides, which can protect the integrity of cell membrane structure and function, and reduce oxidative stress damage to cells [22]. One important function of dandelion is antioxidant activity. In the present study, MDA was a product of lipid peroxidation that can indicate the degree of oxidative damage [23]. SOD, CAT and GSH can maintain a direct balance between oxidants and antioxidants by clearing free radicals [24]. In a mouse model of liver injury, dandelion can normalize the levels of serum MDA, GSH, and SOD in mice [25]. CAT, T-AOC, SOD and GSH-Px are core antioxidant enzymes that eliminate reactive oxygen species, while MDA reflects the degree of lipid peroxidation and oxidative damage. In the present study, the medium dose of DSFP numerically elevated serum antioxidant capacity indices and maintained low MDA concentration, but most antioxidant parameters did not differ significantly among treatments. Thus, these results do not support a general conclusion that DSFP improved systemic antioxidant capacity. However, high-dose DSFP significantly reduced GSH-Px activity, and we speculate that this may be caused by excessive polyphenols interfering with antioxidant enzyme synthesis.

It has been shown that feeding fermented dandelions at a dose of 500 mg/kg results in a higher bursa index at 21 days and a higher spleen index at 42 days [17]. In our study, high-dose DSFP significantly reduced cardiac and renal organ indices, while no significant differences were found in lung, liver, or spleen organ indices. This result indicates that the heart and kidney were more sensitive to high-dose DSFP, and the visceral effect of DSFP displayed tissue selectivity. Both heart and kidney are high-energy-consuming organs. We speculate that the decreased cardiac and renal organ indices induced by high-dose DSFP implied that excessive supplementation might inhibit visceral cell proliferation or disrupt energy distribution in vivo, thereby resulting in the reduction of relative organ weight.

Small intestinal length is a marker reflecting intestinal development and digestion–absorption capacity. DSFP remarkably extended duodenum length, suggesting that dandelion active compounds promote duodenal mucosal proliferation and the early intestinal development of broilers. However, jejunum and ileum length declined with rising DSFP doses, especially in the high-dose group. We speculated that high-concentration bitter polyphenols suppressed excessive proliferation of posterior intestinal tissue. Overall, low and medium DSFP supplementation optimizes small intestinal development by lengthening duodenum without severely shortening jejunum and ileum, thus benefiting nutrient digestion and absorption in broilers.

The intestinal microbiota plays a crucial role in the health and production of poultry [26]. This is considered a biological barrier for innate immunity in the gut, maintaining host immune homeostasis, regulating host metabolism, and fermenting food to promote absorption by the body [27,28]. It has been shown that supplementing the diet with fermented dandelion improves the gut microbiota of broiler chickens [29]. Previous studies had confirmed that plants belonging to Asteraceae can alter the gut microbiota of chickens [30,31]. According to the present study, supplementing dandelion extract could improve the Shannon, Chao1, and ACE indicators of gut microbiota in golden pompano [32]. Previous studies showed that adding dandelion or fermented dandelion to the diet could reduce the alpha diversity of gut microbiota in broiler chickens at 21 days of age [17,29]. In broiler production, it has been reported that the supplementation of dandelion increases the relative richness of Lactobacillus in the ileum and the feed conversion rate [29]. In the production of laying hens, it has been found that supplementation with root extract can increase the relative abundances of Actinomycetes and Firmicutes and is beneficial to *Trachinotus ovatus* [32]. The decrease in gut microbiota diversity might be related to the antibacterial substances of dandelions [10]. Similarly, the differences in β diversity between different groups might also be related to the antibacterial components of dandelion. In this study, alpha diversity results illustrated that only medium-dose DSFP reduced cecal microbial richness and evenness, while low and high doses maintained stable community diversity consistent with the control group. Beta diversity PCoA showed a visual separation among treatment groups, suggesting a possible trend toward microbiota structural differentiation associated with medium-dose DSFP addition; however, the ANOSIM result was not statistically significant, and therefore this pattern should be interpreted as a treatment-associated trend rather than a confirmed treatment effect. Taxonomic abundance profiles revealed that DSFP modified the relative proportion of core phyla Firmicutes and Bacteroidota, especially in the DSFP-M group. LEfSe analysis further identified several taxa that have previously been reported to be associated with short-chain fatty acid production in the medium-dose DSFP group. However, SCFA concentrations were not measured in this study; therefore, whether these taxa actually increased SCFA production remains unknown and requires further investigation. Collectively, low and high doses of DSFP exerted mild regulatory effects without disrupting microbial homeostasis; by contrast, medium-dose DSFP moderately altered the composition of cecal microbiota. Combined with previous intestinal phenotype and antioxidant data, we tentatively hypothesize that moderate DSFP supplementation alters intestinal microecology to improve gut barrier and antioxidant capacity, while excessive microbial filtering at high dosage may counteract partial beneficial physiological effects in broilers. These interpretations should be considered tentative because of the lack of significant overall community-level differences and the absence of direct SCFA measurements.

Adding DSFP increased feed costs in a dose-dependent manner. Nevertheless, moderate DSFP supplementation improved broiler weight gain and sales revenue, fully offsetting extra feed expenses and achieving the maximum gross profit. High-dose DSFP caused excessive feed investment, and its growth-promoting effect was weakened by disturbed intestinal microbiota and reduced antioxidant capacity, failing to further raise economic benefits. Low-dose DSFP slightly elevated profit without statistical significance. Collectively, medium DSFP addition is the optimal level for broiler production from an economic perspective. It should be noted that this economic evaluation was calculated based on feed ingredient and broiler prices at the time of the trial, and the economic outcome may change with market price fluctuations.

## 5. Conclusions

In summary, this study demonstrates that dietary supplementation with dandelion ultrafine powder effectively enhances the growth performance and economic returns of broiler chickens. The beneficial effects may be associated with bioactive compounds from dandelion. Previous studies have reported that ultrafine grinding may facilitate the release of intracellular ingredients trapped in plant cell walls; however, the release, absorption, and bioavailability of these bioactive compounds were not directly measured in the present experiment. These findings collectively suggest that 1.0% DSFP showed the most favorable overall response in improving growth performance, maintaining intestinal health, and optimizing economic benefits among the different DSFP inclusion levels evaluated in this study.

## Figures and Tables

**Table 1:** Composition of the experimental starter and grower diets fed to broilers.

Diets Groups	Starter [g/kg]				Grower [g/kg]			
	CON	DSFP-L	DSFP-M	DSFP-H	CON	DSFP-L	DSFP-M	DSFP-H
Corn	57.20	57.20	57.20	57.20	62.40	62.40	62.40	62.40
Soybean meal	32.50	32.50	32.50	32.50	26.80	26.80	26.80	26.80
Soybean oil	2.50	2.50	2.50	2.50	3.00	3.00	3.00	3.00
Corn gluten meal	2.00	2.00	2.00	2.00	2.00	2.00	2.00	2.00
Limestone	1.20	1.20	1.20	1.20	1.10	1.10	1.10	1.10
Dicalcium phosphate	1.80	1.80	1.80	1.80	1.60	1.60	1.60	1.60
Salt	0.30	0.30	0.30	0.30	0.30	0.30	0.30	0.30
Premix	2.50	2.50	2.50	2.50	2.50	2.50	2.50	2.50
Dandelion	0.00	5.00	10.00	30.00	0.00	5.00	10.00	30.00
Energy Value [MJ] and Essential Nutrients [g/kg] of Experimental Diets								
Metabolizable energy, MJ/kg	12.55	12.49	12.43	12.18	12.90	12.84	12.77	12.52
Crude protein, %	21.50	21.39	21.29	20.87	19.50	19.40	19.31	18.93
Calcium, %	1.00	1.00	0.99	0.97	0.90	0.90	0.89	0.87
Available phosphorus, %	0.45	0.45	0.45	0.44	0.42	0.42	0.42	0.41
Lysine, %	1.10	1.09	1.09	1.07	0.95	0.95	0.94	0.92
Methionine, %	0.50	0.50	0.50	0.49	0.42	0.42	0.42	0.41

**Table 2:** Effects of DSFP on the ADG, ADFI and FCR of broilers (*n* = 6).

Items		Groups			
		CON	DSFP-L	DSFP-M	DSFP-H
1–21 d	ADG/g	17.55 ± 0.61 ^c^	20.75 ± 0.06 ^b^	23.17 ± 1.09 ^a^	16.94 ± 0.66 ^c^
	ADFI/g	30.41 ± 2.65	29.09 ± 1.32	30.42 ± 3.50	29.09 ± 3.50
	FCR/g	1.73 ± 0.11	1.4 ± 0.07	1.32 ± 0.19	1.73 ± 0.08
22–42 d	ADG/g	80.63 ± 4.23 ^b^	90.35 ± 2.10 ^b^	101.42 ± 2.09 ^a^	100.35 ± 3.29 ^a^
	ADFI/g	171.95 ± 17.35	171.95 ± 18.52	190.48 ± 18.33	174.07 ± 6.77
	FCR/g	2.13 ± 0.10	1.92 ± 0.25	1.88 ± 0.16	1.91 ± 0.07
1–42 d	ADG/g	49.4 ± 2.62 ^c^	55.89 ± 0.81 ^b^	62.65 ± 1.90 ^a^	58.98 ± 1.45 ^ab^
	ADFI/g	115.07 ± 7.15 ^b^	114.41 ± 3.03 ^b^	124.34 ± 1.15 ^a^	115.07 ± 3.43 ^b^
	FCR/g	2.21 ± 0.51	2.33 ± 0.43	2.18 ± 0.38	2.50 ± 0.57

CON, control group; DSFP-L, low-dose DSFP group; DSFP-M, medium-dose DSFP group; DSFP-H, high-dose DSFP group. ^a–c^ Means within a row with different superscripts differ significantly (*p* < 0.05).

**Table 3:** Effects of dietary DSFP supplementation on heart, liver, spleen, lung and kidney organ indices of broilers (*n* = 6).

Items	Groups			
	CON	DSFP-L	DSFP-M	DSFP-H
Heart organ index (g/kg)	6.18 ± 0.53 ^ab^	6.42 ± 0.38 ^a^	5.91 ± 0.27 ^ab^	5.28 ± 0.17 ^b^
Liver organ index (g/kg)	27.48 ± 2.81	27.21 ± 1.82	26.91 ± 2.45	21.45 ± 1.14
Spleen organ index (g/kg)	1.59 ± 0.11	1.51 ± 0.23	1.23 ± 0.14	1.35 ± 0.08
Lung organ index (g/kg)	5.96 ± 0.64	6.54 ± 0.38	6.58 ± 0.60	5.36 ± 0.47
Kidney organ index (g/kg)	7.28 ± 0.47 ^a^	6.40 ± 0.31 ^ab^	6.94 ± 0.33 ^ab^	6.07 ± 0.18 ^b^

^a,b^ Means within a row with different superscripts differ significantly (*p* < 0.05).

**Table 4:** Effects of dietary DSFP supplementation on small intestinal segment length of broilers (*n* = 6).

Items	Groups			
	CON	DSFP-L	DSFP-M	DSFP-H
Duodenum length (cm/kg)	6.61 ± 0.19 ^b^	9.61 ± 0.59 ^a^	9.88 ± 0.37 ^a^	9.14 ± 0.43 ^a^
Jejunum length (cm/kg)	26.51 ± 1.00 ^a^	23.79 ± 1.58 ^ab^	25.41 ± 0.14 ^ab^	20.81 ± 1.93 ^b^
Ileum length (cm/kg)	29.88 ± 1.17 ^a^	25.67 ± 1.11 ^b^	27.11 ± 0.65 ^b^	19.96 ± 0.27 ^c^

^a–c^ Means within a row with different superscripts differ significantly (*p* < 0.05).

**Table 5:** Effects of dietary DSFP supplementation on the serum antioxidant indices of broilers (*n* = 6).

Items	Groups			
	CON	DSFP-L	DSFP-M	DSFP-H
GSH-Px (U/mL)	2258.05 ± 13.91 ^ac^	2332.04 ± 35.80 ^a^	2342.35 ± 20.43 ^a^	1828.61 ± 282.07 ^bc^
GSH (mg/L)	2.13 ± 0.32 ^bc^	4.45 ± 1.40 ^ab^	5.65 ± 1.48 ^a^	1.52 ± 0.47 ^c^
T-AOC (mmol/L)	0.52 ± 0.04 ^a^	0.51 ± 0.05 ^a^	0.57 ± 0.12 ^a^	0.48 ± 0.06 ^a^
SOD (U/mL)	101.57 ± 8.50 ^a^	110.21 ± 5.28 ^a^	109.11 ± 2.70 ^a^	98.91 ± 2.11 ^a^
CAT (U/mL)	0.64 ± 0.32 ^a^	1.09 ± 0.32 ^a^	0.49 ± 0.14 ^a^	0.63 ± 0.17 ^a^
MDA (nmol/mL)	40.00 ± 6.05 ^a^	66.86 ± 9.58 ^a^	62.21 ± 13.24 ^a^	42.91 ± 10.97 ^a^

^a–c^ Means within a row with different superscripts differ significantly (*p* < 0.05).

**Table 6:** Effects of DSFP on the production economic performance of broilers (CNY, Chinese Yuan) (*n* = 6).

Items	Groups			
	CON	DSFP-L	DSFP-M	DSFP-H
Feed price (CNY/kg)	2.81	2.89	2.96	3.26
Broiler market price (CNY/kg)	9.12	9.12	9.12	9.12
Total feed cost (CNY/bird)	13.58 ± 0.84 ^c^	13.89 ± 0.37 ^c^	15.46 ± 0.14 ^ab^	15.75 ± 0.47 ^a^
Sales revenue (CNY/bird)	20.74 ± 2.21 ^b^	22.27 ± 1.68 ^ab^	24.49 ± 0.77 ^a^	23.94 ± 1.05 ^a^
Gross profit (CNY/bird)	7.15 ± 3.03 ^b^	8.38 ± 2.00 ^ab^	9.03 ± 0.72 ^a^	8.18 ± 1.40 ^ab^

^a–c^ Means within a row with different superscripts differ significantly (*p* < 0.05).

## Data Availability

The original contributions presented in the study are included in the article. Further inquiries can be directed to the corresponding author.
